# Identification of Marine-Derived SLC7A11 Inhibitors: Molecular Docking, Structure-Based Virtual Screening, Cytotoxicity Prediction, and Molecular Dynamics Simulation

**DOI:** 10.3390/md22080375

**Published:** 2024-08-20

**Authors:** Jiaqi Chen, Xuan Li, Jiahua Tao, Lianxiang Luo

**Affiliations:** 1 The First Clinical College, Guangdong Medical University, Zhanjiang 524023, China; a850978965a@gdmu.edu.cn (J.C.); x.li@gdmu.edu.cn (X.L.); jiahuatao@gdmu.edu.cn (J.T.); 2 The Marine Biomedical Research Institute of Guangdong Zhanjiang, School of Ocean and Tropical Medicine, Guangdong Medical University, Zhanjiang 524023, China

**Keywords:** ferroptosis, SLC7A11, marine natural compound, virtual screening, fragment substitution, molecular dynamics simulation

## Abstract

The search for anticancer drugs that target ferroptosis is a promising avenue of research. SLC7A11, a key protein involved in ferroptosis, has been identified as a potential target for drug development. Through screening efforts, novel inhibitors of SLC7A11 have been designed with the aim of promoting ferroptosis and ultimately eliminating cancer cells. We initially screened 563 small molecules using pharmacophore and 2D-QSAR models. Molecular docking and ADMET toxicity predictions, with Erastin as a positive control, identified the small molecules 42711 and 27363 as lead compounds with strong inhibitory activity against SLC7A11. Further optimization resulted in the development of a new inhibitor structure (42711_11). Molecular docking and ADMET re-screening demonstrated successful fragment substitution for this small molecule. Final molecular dynamics simulations also confirmed its stable interaction with the protein. These findings represent a significant step towards the development of new therapeutic strategies for ferroptosis-related diseases.

## 1. Introduction

Cancer treatment has always been a primary focus of research, with the goal of targeting cancer cells while preserving healthy ones. Regulated cell death (RCD) is essential in managing cancer cell growth and ensuring cellular balance [1]. Ferroptosis, which was identified in 2012, is intricately associated with iron, lipid, and antioxidant metabolism [2]. It is an emerging field of study that has major implications for a range of diseases, such as cancer, ischemic organ damage, neurological disorders, and autoimmune conditions [3]. Recent research indicates that ferroptosis is controlled by a range of factors, particularly iron and lipid metabolism, as well as the equilibrium between oxidants and antioxidants. Various pathways, such as the system XC- glutathione (GSH) glutathione peroxidase 4 (GPX4) pathway, the transsulfuration pathway, the mevalonate pathway (MVA), and the ferric senescence suppressor protein 1 (FSP1)-coenzyme Q10 (CoQ10) pathway, have been identified as regulators of ferroptosis [4]. Despite the ongoing evolution of these findings, researchers have made significant strides in identifying both ferroptosis inhibitors and inducers for potential clinical use. These promising outcomes emphasize the momentum behind current research endeavors. And the aim of this study is to discover promising marine compounds that induce ferroptosis, with the ultimate goal of combatting cancer.

The current findings underscore the significance of studying ferroptosis. For example, Chen et al. illustrated that boosting the levels of the ferroptosis inhibitor glutathione peroxidase 4 (GPX4) could potentially enhance the health and survival of motor neurons in vivo, offering promising therapeutic pathways for neurodegenerative disorders like Alzheimer’s disease [5,6]. It was proposed by Fang et al. that suppressing the Nrf2/Hmox1 axis-induced ferroptosis in DOX might be beneficial for the treatment of cardiomyopathy [7]. Additionally, in 2022, ferroptosis was suggested to play a role in the COVID-19 pandemic; Jankauskas et al. discovered links between COVID-19 severity and changes in serum levels of ferroptosis-related factors in patients [8]. Together, ferroptosis inducers and inhibitors show potential for improving treatment approaches for a wide range of conditions [9].

In addition, the targeted manipulation of iron metabolism is thought to have the potential to strengthen the body’s immune response to tumors and enhance the effectiveness of cancer treatments [10]. Therefore, this research suggests the possibility of creating ferroptosis inducers for treating cancer. The term “ferroptosis” was introduced in 2012, at the same time as Stockwell’s identification of Erastin as the first inducer and the crucial ferroptosis pathway that involves system xc2. Erastin, a small compound, specifically targets cells that carry RAS oncogenic mutations [11]. System xc- is composed of the transporter subunit SLC7A11 (xCT) and the regulatory subunit SLC3A2 (4F2hc), which work together to facilitate the import of cystine into the cell. Once inside the cytoplasm, cystine is converted to cysteine, allowing for the synthesis of glutathione (GSH). This process enables glutathione peroxidase 4 (GPX4) to remove lipid peroxides from cell membranes, helping to prevent ferroptosis [12]. Erastin inhibits system xc-‘s activity, thereby inducing cellular ferroptosis [9]. However, Erastin’s limited water solubility and metabolic instability preclude its direct clinical application [13]. Consequently, there is an urgent need to discover new ferroptosis inducers. Yan et al. employed cryo-electron microscopy (cryo-EM) to analyze a complex involving the ferroptosis inhibitor Erastin bound to the proteins SLC7A11 and SLC3A2 [14]. SLC7A11 serves as a key functional subunit of system xc-. Overexpression of SLC7A11 promotes tumor growth by inhibiting ferroptosis, making it a potential therapeutic target for cancer treatment [15,16,17,18]. This structural analysis provides essential information for the creation of ferroptosis inducers, showcasing its potential as a viable therapeutic target.

As a coastal city, our geographical location uniquely positions us to explore marine resources. Marine natural products exhibit distinctive characteristics absent in terrestrial organisms, including high diversity and biological activity [19], making them promising candidates for discovering new ferroptosis inducers.

Computer-aided drug design (CADD) integrates various computational tools to streamline drug development, offering advantages such as cost reduction and accelerated processes [20]. This study leveraged not only CADD but also fragment-based drug design (FBDD), a promising approach for discovering and optimizing lead compounds [21].

**Figure 1:**
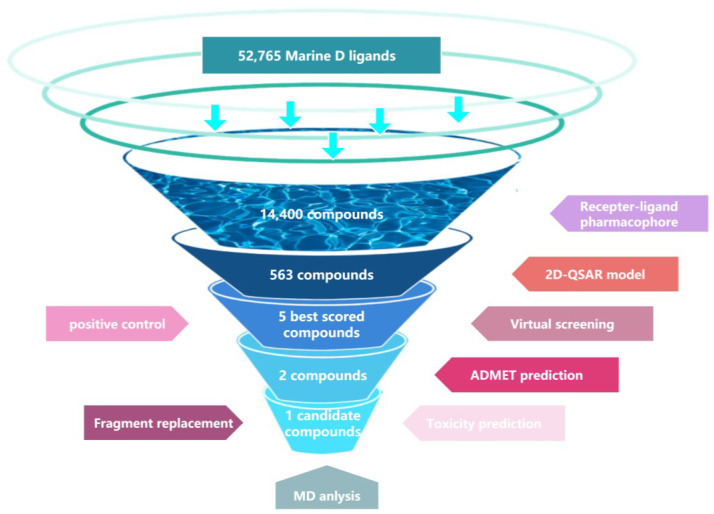
The workflow of this study.

Several studies utilize computer-aided drug design methods to identify potential drug candidates for treating ferroptosis-related diseases from marine compound libraries. For instance, Liao et al. have developed a ferroptosis inhibitor targeting ALOX15 based on two distinct approaches [22,23]. This study supports our current research efforts by focusing on the overall structure of the Erastin-bound xCT-4F2hc complex (PDB ID: 7EPZ) for screening marine natural compounds to find potential SLC7A11 inhibitors. Initially, pharmacophore models were created to identify compounds with desired characteristics, followed by 2D-QSAR modeling to predict compound activity. Molecular docking was then used to evaluate compounds against positive controls, with further optimization through fragment substitution. Toxicity predictions were made to assess clinical applicability, and molecular dynamics simulations were used to confirm protein–ligand binding states and stability. This study identified marine database SLC7A11 inhibitors as promising candidates for inducing ferroptosis in cancer cells. The workflow of this study is illustrated in Figure 1.

## 2. Results

### 2.1. Virtual Screening Based on Pharmacophore

**Figure 2:**
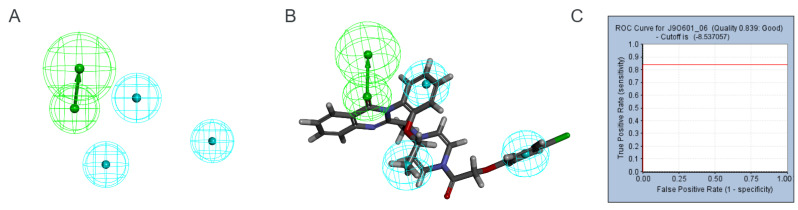
Hydrophobic group features are represented by blue spheres and hydrogen bond donor features are represented by green spheres. (**A**) Pharmacophore_6 pharmacophore. (**B**) Erastin and Pharmacophore_6 recombination effect diagram. (**C**) ROC curve of Pharmacophore_6.

Pharmacophore modeling is a process that involves simulating the active conformation of ligand molecules by conducting conformational searches and molecule superpositions. This enables researchers to deduce and interpret potential interactions between receptor and ligand molecules [24]. This method serves as a crucial tool in the discovery of lead compounds. Using Discovery Studio 2019, we constructed six receptor–ligand-based pharmacophore models (Table 1). Pharmacophore6, validated with a minimum of four features and exhibiting sensitivity, specificity, and ROC curve values exceeding 0.8, was chosen for further investigation (Figure 2). The database comprising 52,765 marine compound molecules underwent preprocessing using the Prepare Ligand module. Subsequently, these molecules underwent screening via Ligand Pharmacophore Mapping in Discovery Studio 2019, with settings including Best Mapping Only (True), Maximum Omitted Feature (0), Fitting Method (Rigid), and Parallel Processing (False). Application of Pharmacophore_6 identified 14,440 molecules that remained in the marine compound database.

### 2.2. Screening Based on the 2D-QSAR Model

#### 2.2.1. Dataset Preparation

**Figure 3:**
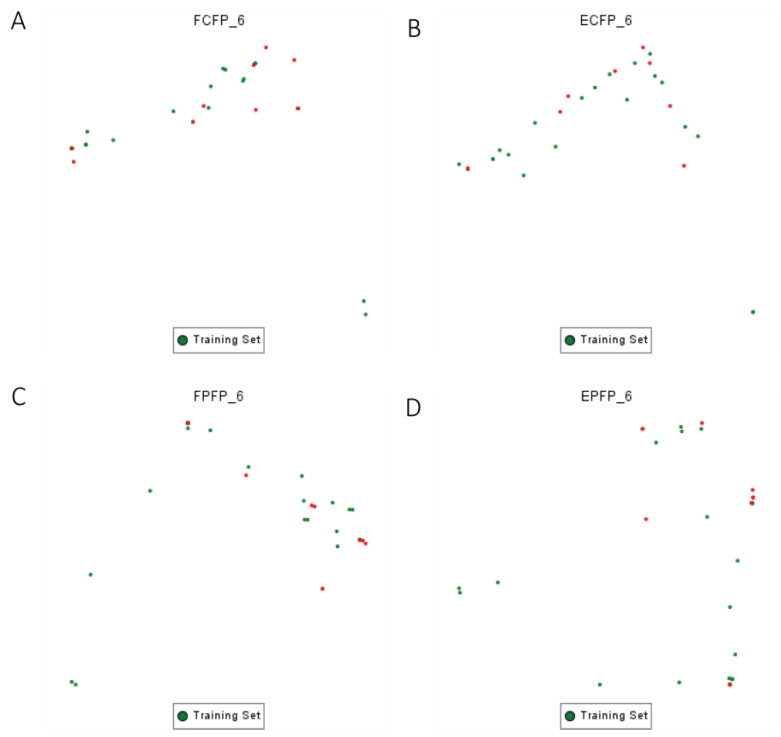
Construction of a 2D-QSAR model using a training set–test set of molecule chemical spatial distributions. Training set molecules are represented in green, while test set molecules are represented in red. (**A**) Chemical spatial distribution calculated based on FPFP_6 (Functional classed Extended Connectivity Fingerprint_6); (**B**) chemical spatial distribution calculated based on EPFP_6 (Atom type PathBased Fingerprint_6); (**C**) chemical spatial distribution calculated based on FCFP_6 (Functional classed Extended Connectivity Fingerprint_6); (**D**) chemical spatial distribution calculated based on ECFP_6 (Atom type Extended Connectivity Fingerprint_6).

We partitioned the 31 target inhibitors into training and test sets using a 7:3 ratio. For each molecule in both sets, we computed FPFP/EPFP/FCFP/ECFP_6 fingerprint profiles separately to assess their distribution across chemical space and overlap. These descriptors were utilized to characterize each molecule in a multidimensional property space, facilitating the visualization of their distributions across specific spatial coordinates. The findings are illustrated in Figure 3A–D. Analysis of the chemical spatial distribution based on fingerprint features reveals a consistent pattern between the training and test sets, indicating no significant bias in physicochemical properties due to the random dataset division.

#### 2.2.2. Two-Dimensional-QSAR Model Construction, Testing and Filter

**Figure 4:**
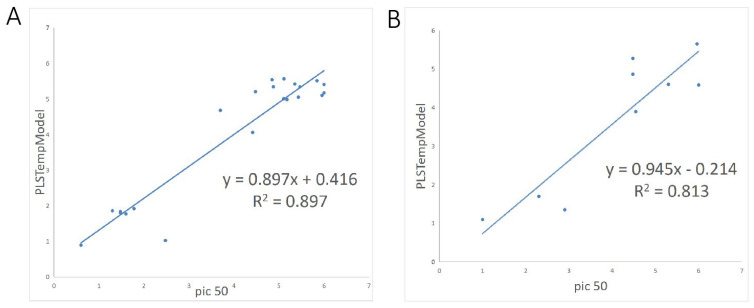
(**A**,**B**) Linear regression equations for the training and test sets of the constructed 2D-QSAR model.

For the construction of molecular descriptors in our 2D-QSAR model, we utilized default descriptors such as ALogP, Molecular_Weight, Num_AromaticRings, Num_H_Acceptors, Num_H_Donors, Num_Rings, Num_RotatableBonds, and Molecular_FractionalPolarSurfaceArea, which incorporates molecular fingerprints. Detailed characterization of these molecular properties is provided in Table S1 in the Supplementary Material. Molecular fingerprints are recognized as critical metrics within these descriptors [25]. Among the options available in the Fingerprints section of Calculable Properties, ECFC_6, ECFP_6, and EPFC_6 were selected as the descriptors for molecular fingerprints after comparison, resulting in the model demonstrating excellent R^2^ compared to alternative molecular fingerprints (Figure 4). The final R^2^ values were 0.897 for the training set and 0.813 for the test set, indicating robust predictive capability.

Using the 2D-QSAR model developed above, we screened the remaining 14,400 small molecules using the Calculate Molecular Properties module of the Discovery Studio 2019 platform. We selected small molecules predicted to have activity above 4.5 nmol, resulting in 563 small molecules chosen for the subsequent phase of the study.

### 2.3. Structure-Based Virtual Screening

Based on the previous step, we applied the established 2D-QSAR model to predict the bioactivity of small molecules. We identified 563 small molecules with predicted bioactivity values exceeding 4.5 nmol. These molecules were initially prepared in 1665 conformations using the Prepare Ligand module of Discovery Studio 2019, while the protein underwent preparation using the prepare protein module. Yan et al. identified protein residues Gln191, Phe254, and Phe336 as pivotal for mediating interactions with the positive compound Erastin [14]. We adopted this information as a reference for docking the candidate compounds using the Libdock docking method, targeting the aforementioned residues as active sites (center coordinates 146.527, 145.684, 120.68, and radius 14.135). The docking score served as our primary screening criterion.

First, we isolated the protein and ligand from protein 7EPZ. Subsequently, the LibDock module of Discovery Studio 2019 performed the docking simulations. Utilizing the software’s predicted active site function, we identified the active site generating the highest number of docking poses, with center coordinates of 125.945, 123.717, and 113.296, and a radius of 10.7. The highest docking score obtained was 137.656, selected as the screening criterion for the Libdock score; small molecules surpassing this threshold were deemed suitable for further investigation.

Secondly, Erastin served as a positive control for Libdock, yet its docking score (88.9923) fell short of the proto-ligand proteins’ docking score mentioned earlier as a screening criterion. To identify compounds with superior scores, we continued to use the proto-ligand photoprotein docking score as a screening criterion.

Through a dual screening process involving the Erastin positive control and proto-ligand photoprotein docking, we identified small molecules with docking scores higher than both criteria for the subsequent study phase. Each small molecule adopted a distinct conformation, and only the highest-scoring conformer from each was selected, yielding five small molecules for further analysis. Subsequent fine docking of these five small molecules against Erastin confirmed favorable docking scores. Table 2 presents the structural and docking specifics of the positive compound Erastin and the five screened compounds.

### 2.4. Predictive Analysis of Toxicity for Swissadme

To evaluate the potential of the five compounds identified in the previous screening for clinical efficacy, we employed the online tool Swissadme to predict their in vivo absorption, distribution, metabolism, excretion, and toxicity profiles. Parameters such as the oil–water partition coefficient (logP), gastrointestinal absorption, water solubility, and blood–brain barrier permeability were discussed, and their specific values are detailed in Table 3. All compounds exhibited notably high gastrointestinal absorption rates, indicating their suitability for oral administration in clinical dosing regimens.

Lipinski’s Rule of Five is a widely utilized criterion in virtual screening. According to Lipinski’s guidelines, molecular weight should be below 500; the number of hydrogen bond donors should not exceed 5; the number of hydrogen bond acceptors should not exceed 10; the compound should have fewer than a specified number of rotatable bonds; and the oil–water partition coefficient (LogP) should be ≤5 [26]. However, three of the small molecules (64778, 43932, 41093) did not conform to these criteria.

The oil–water partition coefficient (LogP) represents the logarithmic ratio of a substance’s partition coefficients between octanol and water. Swissadme provides five algorithms to calculate LogP, and we computed their average for comparative analysis. A higher LogP indicates greater lipophilicity, while a lower LogP suggests higher hydrophilicity. According to predictions, all five compounds exhibit poor to moderate water solubility. Moderately water-soluble compounds maintain lower LogP values (around 2), whereas poorly water-soluble ones have higher LogP values (around 4). This inverse relationship between water solubility and LogP values is evident across these small molecules.

Small molecules typically enter the brain via passive diffusion, and their blood–brain barrier penetration correlates partly with their lipophilicity. Among the compounds studied, only 64,778 was predicted to penetrate the blood–brain barrier, possessing the highest LogP value.

In conclusion, based on the aforementioned analyses, we selected two small molecules (27363, 42711) for further evaluation.

**Figure 5:**
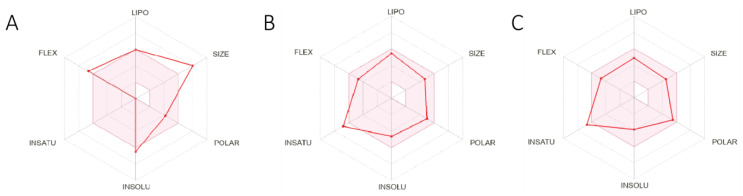
The Swissadme radar plot of the positive control Erastin and 2 lead compounds. (**A**) Radar plot of positive control Erastin. (**B**) Radar plot of lead compound 27363. (**C**) Radar plot of lead compound 42711.

Figure 5 depicts radar plots illustrating six physicochemical properties: lipophilicity, size, polarity, solubility, flexibility, and saturation [27]. The radar plots of these two lead compounds demonstrate that all six properties fall within the pink area of the figure, contrasting with Erastin. This suggests that these compounds may possess more advantageous drug-like properties in terms of bioavailability.

### 2.5. Optimization of Fragment Replacement of Candidate Lead Molecules

To explore further structural optimization of the compounds, we conducted fragment substitution on two small molecules identified from the aforementioned screening. Initially, we examined the 2D interaction diagrams of candidate compounds 27363 and 42711 with protein 7EPZ, focusing on fragments showing minimal interactions with the protein for substitution. Post-substitution, we generated 97 new small molecules for 27363 and 88 for 42711. Subsequently, we performed libdock molecular docking, excluding compounds with docking scores lower than those of the original molecules. Ultimately, this process yielded 2 new small molecules for 27363 and 42 for 42711. Detailed structures and docking scores can be found in Tables S2 and S3 of the Supplementary Material.

### 2.6. Re-Screening of the Optimized Compounds

We screened a total of 44 small molecules post-optimization for toxicity predictions using SwissADME and CLCpred 2.0.

#### 2.6.1. Swissadme

Lipinski’s Rule of Five (RO5) provides physicochemical guidelines for screening potential drug molecules: compounds should have a molecular weight less than 500 g/mol, no more than five hydrogen bond donors (HBDs) and ten hydrogen bond acceptor (HBA) sites, no more than ten rotatable bonds (RBs), a polar surface area (PSA) less than 140 Å, and a LogP value less than 5, indicating low hydrophobicity [26]. A comparison between our candidate compound and the positive control Erastin revealed more RO5 violations with Erastin. Consequently, despite its identification as a ferroptosis inducer long ago, we have refrained from clinical use of Erastin. Few of our candidate compounds adhere strictly to these principles. Moreover, while some small molecules meet Lipinski’s guidelines, their overall drug properties remain unsatisfactory. For instance, in medicinal chemistry, the structural fragment acyclic_C=C-O raises concerns. This fragment persists within the small molecule itself without undergoing optimization through fragment substitution. Its retention is justified by its effective protein interaction, which led us to retain the molecule despite the warning signal. Subsequently, we selected 17 small molecules as candidates for the next phase of cytotoxicity prediction. Detailed results regarding Lipinski’s criteria for these compounds are provided in Table 4.

#### 2.6.2. CLCpred

In vitro cell line cytotoxicity assays are widely employed in experimental studies to evaluate potential antitumor drugs and assess drug safety during development [28]. Given that ferroptosis is implicated in various diseases, particularly cancer, we utilized CLCpred cytotoxicity prediction to re-evaluate the aforementioned 17 small molecules. Our analysis primarily focused on cytotoxicity predictions derived from CHEMBL and PubChem datasets (comprising 128,545 structures), assessing their impact on 391 tumor and 47 normal human cell lines, alongside a panel of NCI60 tumor cell lines.

In Tables S4–S7 of the Supplementary Material, we identified two small molecules (42711_11, 42711_38) from the initial set of 17 that exhibited heightened cytotoxicity against both tumor cells and the NCI60 panel at 10 nm and 100 nm thresholds. Additionally, we calculated the invariant accuracy of prediction (IAP), correlating numerically with the ROC AUC value, and found their predicted IAP values for each cytotoxicity to be above 0.8, indicating robust predictive capability.

Based on these findings, we selected two small molecules with superior docking scores compared to their original counterparts, demonstrating enhanced drug-like properties, particularly in comparison to the benchmark compound Erastin.

As indicated in Table 5, the positive control Erastin exhibits fewer protein interactions compared to compound 42711 and its derivatives. This difference may arise because Erastin’s more folded structure limits available space for small molecules to interact with protein residues. Additionally, for the original compound 42711 and the new molecules 42711_11 and 42711_38 generated through fragment substitution (Table 5), it is evident that they exhibit new interactions in addition to retaining their original ones. For instance, in 42711_11, the substituted fragment forms a halogen bond with TYR-97, while the unaltered tail of the molecule also forms a halogen bond with SER-107. The former likely occurs due to atoms in the replaced fragment forming a halogen bond with the protein, while the latter suggests that the new molecule’s formation alters the conformation of remaining fragments, positioning the benzene ring closer to nearby SER-107 for halogen bonding. In small molecule 42711_38, we observed that the interaction with TYR-113 in the substituted fragment shifted from a hydrogen bond in the original compound to a carbon–hydrogen bond. Concurrently, it developed a more stable interaction with SER-107. Moreover, the hydrogen bond involving PHE-250, originally positioned centrally in the compound, relocated to the opposite side in small molecule 42711_11. These structural alterations in the molecule may induce folding within the fragment, thereby expanding the contact surface area between the small molecule and the protein, facilitating increased interactions with 42711_38.

### 2.7. Molecular Dynamics Simulation and Analysis

**Figure 6:**
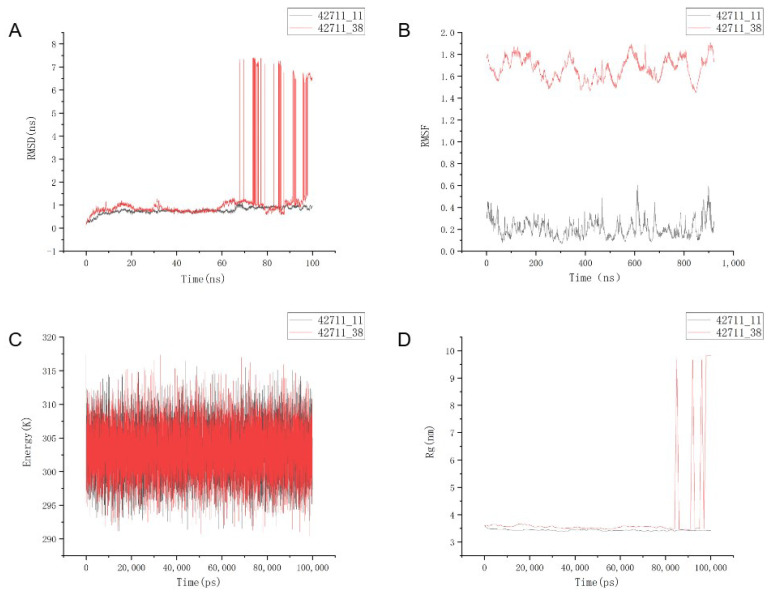
Molecular dynamics simulations of two candidate molecules in complex with a protein. (**A**) root-mean-square deviation fluctuations of the ligand; (**B**) root-mean-square fluctuations of the protein residues forming the complex; (**C**) potential energy fluctuations of the complex system; and (**D**) protein radius of gyration.

In Figure 6A, the 100 ns molecular dynamics simulation reveals significant differences in the stability of conformational fluctuations between two molecules. Specifically, RMSD fluctuations for 42711_11 consistently remained low, ranging between 0.1 and 1.0 nm, indicating stability post-binding to the protein. Conversely, 42711_38 exhibited lower initial fluctuations over the first 60 ns, maintaining stability; however, its RMSD sharply spiked in the latter half of the simulation, potentially due to backbone structure disruption. Ultimately, only 42711_11 achieved full conformational stability.

Subsequently, the root-mean-square fluctuations (RMSFs) of residues in the receptor protein segment of the complex were analyzed to assess ligand–receptor binding stability, as depicted in Figure 6B. Initially, notable differences in RMSF trends between the two ligand–receptor complexes were observed. The notably high RMSF value of 42711_38 suggests significant structural damage during simulation. In contrast, RMSF values for 42711_11 and the protein remained predominantly within the 0.1 to 0.5 range throughout, indicating excellent receptor conformational stability throughout the simulation process.

Additionally, we computed and assessed the radius of gyration (Rg) of the receptor protein structure within the complex system, alongside evaluating the total energy variation of the system. The fluctuation in complex potential energy is illustrated in Figure 6C. Throughout the dynamic simulation, the total potential energy of all small-molecule ligand–protein receptor complexes remained stable, maintaining an estimated average total energy of 303 kJ/mol. This observation supports the stable binding of the novel ligands to the proteins from an energetic perspective. The radius of gyration (Rg) serves as an indicator of the stability and compactness of the protein structure. As depicted in Figure 6D, the average Rg value for proteins in the 42711_11 complex system remained approximately 3.5 nm, indicating that the structure of the protein complexes remained compact throughout the simulation.

**Figure 7:**
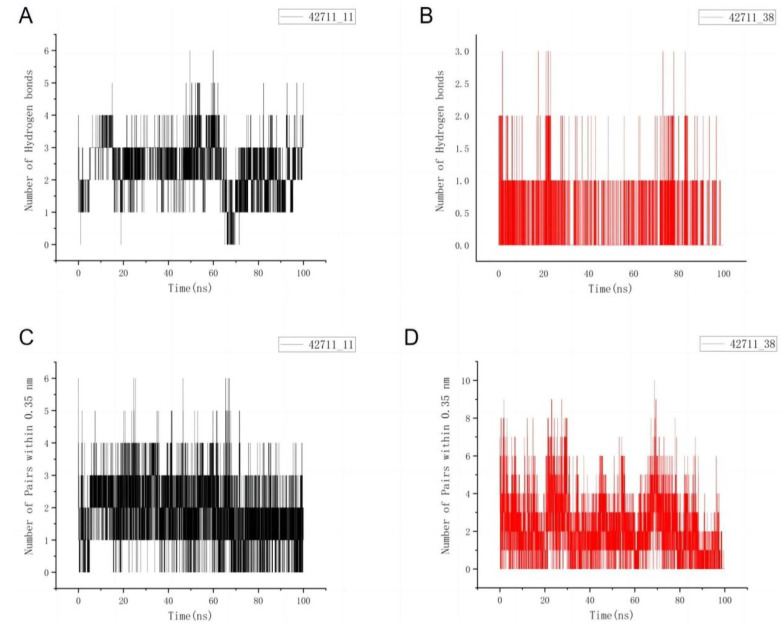
Interaction of two molecules during the simulation. (**A**) Number of hydrogen bonds between 42711_11 and protein. (**B**) Number of hydrogen bonds between 42711_13 and protein. (**C**) Number of interactions between 42711_11 and protein. (**D**) Number of interactions between 42711_13 and protein.

Additionally, we calculated the interaction profile and number of hydrogen bonding interactions within the system. Figure 7A,B illustrate that the 42711_11 molecule exhibited more hydrogen bonding interactions throughout the simulation compared to 42711_38. Hydrogen bonds are robust interactions indicative of binding stability, typically involving atoms within 0.35 nm distance. As depicted in Figure 7C,D, both molecules interact extensively, with 42711_38 showing slightly more interactions. This could explain why 42711_38 performed less effectively in overall molecular dynamics simulations despite having a higher docking fraction—it forms more close interactions but lacks strong hydrogen bonds.

In summary, we have identified 42711_11 as a promising candidate small molecule.

## 3. Discussion

Ferroptosis is currently a prominent area of research with potential implications for treating various diseases, including cancer [2]. Among the pathways implicated in ferroptosis pathogenesis, SLC7A11 stands out as significant [15,16,17,18]. Although Erastin serves as a prototypical ferroptosis inducer, clinical application remains limited due to its suboptimal drug-like properties [13]. Consequently, targeting SLC7A11 holds promise for developing new ferroptosis inducers aimed at treating a broad spectrum of diseases.

Our study began with the development of a pharmacological model exhibiting high sensitivity, specificity, and ROC curve values, the model enabled the screening of 14,440 compounds with ferroptosis-inducing properties from a marine compound library comprising 52,656 small molecules. Unfortunately, we identified fewer target inhibitors in ChEMBL, which may have limited the full characterization of the pharmacophore. This limitation also constrained our subsequent 2D-QSAR modeling approach, making it relatively simple and conventional. However, the model proved reliable and effectively predicted the biological activity of the compounds. Consequently, we selected 563 small molecules with bioactivity greater than 4.5 nmol for the next step of molecular docking. Our target protein (PDB ID: 7EPZ), introduced by Yan et al. through cryo-electron microscopy, is novel. The literature details residues suitable for docking small molecules and proteins, enabling us to identify the active site for docking [14]. Interestingly, the original ligand for this protein is Erastin. Therefore, in this study, Erastin was used as a positive control for subsequent steps, including molecular docking, ADMET toxicity prediction, and fragment substitution (Figure 5, Table 2 and Table 5). During molecular docking, we utilized double scoring with Libdock and CDOCKER to identify five lead compounds from 563 small molecules. ADMET toxicity prediction then eliminated three compounds with undesirable drug properties. Fragment substitution was conducted on the remaining two small molecules (42711, 27363), resulting in the generation of 185 new compounds. Molecular docking and ADMET toxicity prediction were repeated with the original compounds (42711, 27363) and Erastin as a positive control, leading to the identification of 17 small molecules. CLCPred analysis was performed next, selecting two compounds (42711_11, 42711_38) that exhibited higher toxicity to tumor cells. Finally, molecular dynamics simulations identified one compound (42711_11) with stable interactions with proteins.

However, this small molecule did not show superior results in cytotoxicity prediction, despite being better than Erastin. This outcome may be due to targeting a complex bound to Erastin, a compound known for its poor pharmacophore properties. In addition, we referenced the three residues of Erastin that interact with the target, based on the docking analysis of the small molecule with the protein, as described in a previous study [14]. Thus, our screening approach may preferentially identify marine compounds structurally similar to Erastin, as structural similarity often correlates with functional properties. Notably, the Swiss analysis also identified partial structural alerts in medicinal chemistry reports, reinforcing this perspective. However, virtual screening can only predict the physicochemical binding affinity of a molecule, not its intrinsic activity against the target protein, which must be validated by subsequent in vitro experiments.

Notably, there have been no previous instances of discovering ferroptosis inducers using these target proteins in a computer-aided drug design framework. In this study, we utilized a library of marine compounds known for their high biological activity to explore potential drug candidates. Fragment substitution was employed to optimize and innovate small molecules further. Additionally, we employed CLCpred to predict cytotoxicity, aiding in the screening of small molecules that could potentially induce ferroptosis across various diseases, particularly in cancer. With dual toxicity predictions from SwissADME and CLCpred, this finding offers evidence supporting the derivation of small molecules possessing enhanced drug-like properties.

It is noteworthy that, besides Erastin, another ferroptosis inducer has sparked new ideas. In 2020, Wang et al. introduced a modified ferroptosis inducer known as IKE [29]. Derived from Erastin, IKE effectively induces ferroptosis by inhibiting system Xc and obstructing cystine input [30]. IKE exhibits greater metabolic stability compared to Erastin [31], suggesting a promising avenue for further exploration of ferroptosis inducers.

## 4. Materials and Methods

### 4.1. Preparation of the Protein and Small-Molecule Datasets

The protein with PDB ID 7EPZ, titled “Overall structure of Erastin-bound xCT-4F2hc complex”, was obtained from the RCSB PDB database (https://www.rcsb.org/, accessed on 22 March 2024). Protein preparation was conducted using the protein module of Discovery Studio 2019. The prepared protein molecules were utilized for screening SLC7A11 inhibitors.

In this study, a total of 52,765 marine natural compounds were collected from the Seaweed Metabolite Database (SWMD) (http://www.swmd.co.in, accessed on 1 March 2024), Marine Natural Products Database (CMNPD) (https://www.cmnpd.org/, accessed on 1 March 2024), and Marine Natural Product Database (MNP) (http://docking.umh.es/, accessed on 1 March 2024).

### 4.2. Compound Preparation to Construct a Pharmacophore Model

ChEMBL is an open database that houses binding, functional, and ADMET information on numerous drug-like bioactive compounds [32]. We identified 47 inhibitors of SLC7A11 with IC_50_ values from CHEMBL (https://www.ebi.ac.uk/chembl/, accessed on 18 May 2024). To ensure continuity in our investigations, molecules with incomplete data were excluded, resulting in a final set of 31 compounds, detailed in Table S8 in the Supplementary Material.

Erastin, known for its ability to inhibit the xCT-4F2hc complex by blocking cystine import, depleting intracellular glutathione (GSH), and hindering cystine–glutamate exchange [14], was chosen as our positive control. Additionally, Erastin was subjected to DUD-E (https://dude.docking.org/, accessed on 19 May 2024) to generate 51 decoy molecules for future pharmacophore studies.

### 4.3. Pharmacophore Construction and Validation

The receptor protein xCT-4F2hc complex (PDB ID: 7EPZ) was retrieved from the Protein Data Bank RCSB PDB (https://www.rcsb.org/, accessed on 18 May 2024). Given Erastin’s effective inhibition of xCT-4F2hc, we developed a pharmacophore model based on this interaction. In Discovery Studio 2019, Erastin’s binding site served as the active center, with a radius of 11, and the xCT-4F2hc complex underwent preprocessing using Prepare Protein.

For pharmacophore modeling, we utilized the Receptor-Ligand Pharmacophore Generation module in Discovery Studio 2019, configuring Maximum Pharmacophores as 10, Minimum Features as 4, Maximum Features as 6, water Molecules as False, Parallel Processing as False, and Validation as True. The validation process encompassed the 31 SLC7A11 inhibitors as Active Ligands and the 51 decoy molecules as Inactive Ligands.

In the validation results, sensitivity (SE) denotes the model’s capacity to identify active molecules, while specificity (SP) indicates its ability to recognize inactive ones. Higher SE and SP scores signify stronger discriminative capabilities of the pharmacophore model between active and inactive compounds. The ROC curve graphically depicts these metrics: the *x*-axis represents the false positive rate, the *y*-axis represents the true positive rate, and the area under the curve (AUC) quantifies overall performance. The reported quality of 0.500 atop the graph corresponds to the AUC, which ideally exceeds 0.5; greater values indicate enhanced model discriminatory power [33].

### 4.4. Compound Preparation for Constructing the 2D-QSAR Model

For constructing the 2D-QSAR model, we utilized the same SLC7A11 inhibitor as in the pharmacophore model construction. We randomly allocated these 31 small molecules into training and test sets (22 in the training set; 9 in the test set) at a 7:3 ratio using the Generate training and test set module in Discovery Studio 2019. The training set was employed for model training, while the validation set aimed to prevent model overfitting. During this phase, we computed the distribution of each small molecule within the chemical space defined by four molecular fingerprints (FCFP/ECFP/FPFP/EPFP_6). This analysis ensures that the small molecules assigned to either the training or test set exhibit uniform physicochemical properties.

### 4.5. The Construction of 2D-QSAR and Its Validation

QSAR models are employed for predicting the biological activity of compounds. Two-dimensional QSAR specifically involves developing models using 2D descriptors, which facilitate straightforward mathematical calculations to predict the bioactivity of small molecules [34]. In our QSAR modeling approach, we utilize the Partial Least Squares (PLS) method to construct the model. PLS represents a viable alternative to traditional methods such as multiple linear regression and principal component regression [35]. This method employs linear combinations (referred to as components or latent variables) of the original independent variables (X) to maximize the covariance with the dependent variable (Y). PLS conducts regression analyses by correlating observed attribute values with these latent variables, which are linear combinations of the input descriptors from the original model. This approach elucidates interactions between descriptors by quantifying their contributions to the latent variables [36].

The variables used to characterize chemical structures in QSAR encompass diverse properties known as molecular descriptors [37]. Typically, these descriptors are compositional. In this study, we employed specific molecular descriptors including ALogP, MolecularWeight, NumAromaticRings, NumHAcceptors, NumHDonors, NumRings, NumRotatableBonds, and MolecularFractionalPolarSurfaceArea. Additionally, molecular fingerprinting was considered a pivotal descriptor [38]. For optimal predictive performance, we ultimately selected ECFC6, ECFP6, and EPFC6 as molecular fingerprint descriptors following comparative analysis. These molecular descriptors were utilized for constructing the 2D-QSAR model on the segmented training set.

For the constructed 2D-QSAR models, several internal tests evaluate their fitting ability, stability, and internal prediction capability. The primary criterion among these is the correlation coefficient (R^2^) (Equation (1)). A higher R^2^ signifies superior fitting ability of the model.

(1) $$R=\sqrt{1-\frac{\sum {\left(y_{pred}-y_{exp}\right)}^{2}}{\sum {\left(y_{exp}-y_{mean}\right)}^{2}}},$$

### 4.6. Validation of the 2D-QSAR Model

In the absence of an external dataset, validating the predictive power of a model involves conducting statistical external validation [39]. Accordingly, we evaluated the model’s fitting ability, stability, and internal prediction capability using a Leave-One-Out (LOO) cross-validation approach on a divided test set.

The interaction test proceeds by sequentially selecting one sample from N samples, establishing the constitutive relationship with the remaining N-1 samples, and then using the resultant model to predict the activity of the selected sample. This process repeats until all samples have been drawn and predicted. We subsequently calculated the Prediction Error Sum of Squares (PRESS) for the internal test set (Equation (2)), along with the interaction test’s correlation coefficient (Q_LOO_) (Equation (3)).

(2) $$PRESS=\sum {\left(y_{pred}-y_{exp}\right)}^{2},$$

(3) $$Q_{LOO}=\sqrt{1-\frac{PRESS}{\sum {\left(y_{exp}-y_{mean}\right)}^{2}}}$$

The primary parameters used to assess the predictive ability of the 2D-QSAR model are the correlation coefficient R (Equation (1)) and the interaction test correlation coefficient (Q_LOO_) Q^2^ (Equation (3)). Generally, a model is considered to have good predictive ability when R^2^ > 0.8 or Q^2^ > 0.5. The results from the external test set should ideally align with those from internal cross-validation (Q). A 2D-QSAR model was constructed to predict the activities of small molecules for subsequent screening.

### 4.7. Structure-Based Virtual Screening

Molecular docking plays a pivotal role in drug discovery by facilitating the identification of novel therapeutically relevant compounds and predicting molecular-level interactions between ligands and targets [40]. In this study, we employed the LibDock module of the Discovery Studio 2019 platform to conduct molecular docking of 7EPZ and 3552 small molecules sourced from marine natural products. The active sites were delineated based on key residues identified in the 7EPZ-binding pocket as reported by Yan et al. [14].

LibDock evaluates binding capacity and affinity by assessing the interaction between molecules and proteins. The docking process begins with the calculation of a hot zone map for the receptor’s active site, encompassing both polar and nonpolar regions. Subsequently, ligand molecules in various conformations are rigidly aligned individually onto this hot zone map to optimize interactions. Following energy optimization, docking conformations with higher scores are selected and analyzed. Each successful docking conformation is evaluated based on the quantity and type of ligand–receptor interactions observed.

### 4.8. Swissadme Property Screening

SwissADME is a freely accessible web tool designed for assessing the pharmacokinetics, drug similarity, and medicinal chemistry viability of small molecules [27]. It aids in identifying small-molecule drugs better suited for clinical applications. Our focus was on further refining the selection of candidate compounds using Lipinski’s Rule of Five (RO5) as criteria within SwissADME.

Lipinski’s RO5 provides guidelines for screening potential drug molecules based on specific physicochemical properties: molecular weight less than 500 g/mol, no more than five hydrogen bond donor (HBD) sites, no more than ten hydrogen bond acceptor (HBA) sites, no more than ten rotatable bonds (RBs), polar surface area (PSA) less than 140 Å, and a logarithm of the partition coefficient (logP) value less than 5, indicative of hydrophobicity [26]. From the initial pool of five compounds, we evaluated their adherence to these criteria.

### 4.9. Fragment Substitution of the Lead Compound

Fragment substitution-based drug design was employed to optimize lead compounds [21]. To enhance compound quality, the fragment replacement module of Discovery Studio 2019 was utilized for optimization. Following the docking phase, fragments exhibiting fewer interactions within small molecules were selected for replacement. Subsequently, these newly derived small molecules underwent libdock against the target protein, with preference given to those demonstrating superior docking scores compared to the original compounds. Calculations utilized Discovery Studio 2019’s default fragment library, comprising 1,495,478 fragments. Fragment similarity assessments incorporated properties such as the number of rings/aromatic rings and molecular surface area. Molecular structure substitution prioritized fragments with the highest physicochemical resemblance to the originals. Pareto ranking evaluated interactions involving protein formation, Lipinski rule violations, receptor impact, and fragment “novelty”, the latter characterized by chain assemblies, double and aromatic bonds, and aligned N, S, and O atoms. Finally, molecules were reattached to the target using the Libdock module, and the best-performing new molecule was selected based on docking score.

### 4.10. CLC-Pred

CLC-Pred 2.0 (way2drug.com, accessed on 19 June 2024) is a website designed for predicting the cytotoxicity and molecular mechanisms of drug-like compounds in human cell lines, facilitating predictions based on structural formulas across 391 cancer and 47 normal cell lines [28]. Small molecules were submitted sequentially in SMILES format to CLC-Pred for cytotoxicity prediction. The results provided cytotoxicity predictions for compounds across the specified cell lines. The NCI-60 cell line panel, historically used for screening antitumor drugs [41], assessed the toxicity of small molecules at three thresholds: TDP NCI-60 (1 nm), TDP NCI-60 (10 nm), and TDP NCI-60 (100 nm).

Additionally, prediction reliability was evaluated using the mean accuracy (AUC), where AUC > 0.8 indicates reliable predictions. Given this study’s focus on identifying potential inducers of ferroptosis, particularly relevant in various diseases including cancers, molecules exhibiting toxic effects on a broader range of tumor cells were considered to hold greater potential as ferroptosis inducers.

### 4.11. Molecular Dynamics Analysis

Molecular dynamics simulations (MD) are commonly employed to assess the stability of protein–ligand binding systems under specific environmental conditions such as temperature, pressure, and salt solutions, which simulate organismal conditions and incorporate more variables than mere molecular dynamics analysis alone. Our study further analyzed these systems by computing conformational fluctuations in complexes formed by two lead molecules binding to the target over a 100 ns timeframe. The dynamic binding potentials of all candidate compounds were evaluated based on conformational fluctuations, solvent-accessible surface area of ligands, protein radius of gyration, and binding potential. Initially, PDB files of receptor proteins and small molecule ligands were generated and exported from the Discovery Studio platform. Ligand topologies were created using the GAFF force field via Tian Lu’s Sobtop (Version 1.0(dev4), http://sobereva.com/soft/Sobtop, accessed on 24 May 2024). Protein topology files were constructed using the 2019 edition of GROMACS with the AMBER99SB-ILDN force field and TIP3 water model applied for this purpose [42]. A cubic box with a side length of 2.4 nm was constructed to contain the topological model of the protein–receptor complex, and it was filled with SPC216 water molecules to simulate an aqueous environment. The system’s charge neutrality was ensured by adding appropriate quantities of sodium and chloride ions. Initial energy minimization calculations were conducted at a simulation temperature of 300 K over 50,000 steps. Subsequently, equilibration was performed on the receptors, ligands, and solvents within the system under both constant temperature and volume (NVT) and constant temperature and pressure (NPT) conditions, each for 25 ps with a step size of 25,000 steps. Finally, MD simulations were carried out for 100 ns. Analysis included assessment of the root-mean-square deviation (RMSD) and root-mean-square fluctuation (RMSF) of atomic positions, as well as determination of the radius of gyration (Rg), total potential energy variation, and number of hydrogen bonds for each system.

## 5. Conclusions

In this study, we employed computer-aided drug design to screen for ferroptosis inducers targeting SLC7A11 within a marine compound library comprising 52,725 small molecules. Initial screening involved constructing pharmacophore and 2D-QSAR models, followed by molecular docking for high-throughput analysis. ADMET toxicity prediction was also utilized to assess clinical suitability. Fragment substitution of compounds was performed to optimize molecular docking and toxicity prediction. Molecular dynamics simulations ultimately demonstrated promising applications for the lead compound.

In conclusion, our study outlines pathways for discovering ferroptosis inducers, potentially beneficial for treating a broad range of diseases associated with this process. Future research efforts should focus on further exploring ferroptosis inducers to identify additional drug candidates for related diseases.

## Figures and Tables

**Table 1:** Six pharmacophore model-specific values generated using 31 target inhibitors.

Pharmacophore	Number of Features	Feature Set	Sensitivity	Specificity	ROC Curve
Pharmacophore_1	5	AHHHH	0.58065	0.98039	0.516
Pharmacophore_2	4	HHHH	0.58065	0.98039	0.548
Pharmacophore_3	4	AHHH	0.83871	0.98039	0.548
Pharmacophore_4	4	AHHH	0.70968	0.98039	0.944
Pharmacophore_5	4	AHHH	0.87097	0.98039	0.710
Pharmacophore_6	4	AHHH	0.87097	0.98039	0.839

**Table 2:** Structure, Libdock, and CDOCKER energy scores of the five lead molecules and the control inhibitor Erastin.

Molecule	Structure	Libdock Score	Cdocker Energy	CDOCKER Interaction Energy
Erastin	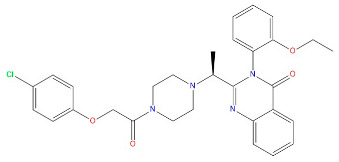	88.9923	21.8788	49.5357
64778	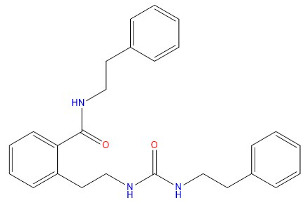	142.415	38.6731	40.911
43932	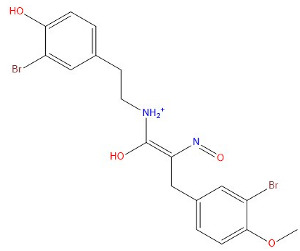	164.288	39.3041	48.0556
41093	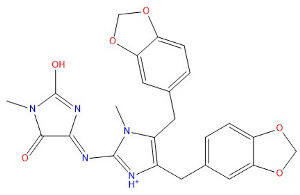	140.318	−10.0613	42.0475
27363	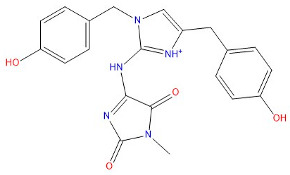	160.383	21.2091	38.5554
42711	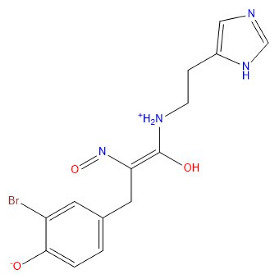	154.262	41.1949	47.8738

**Table 3:** The ADME prediction results for the five lead compounds.

Molecular	Molecular Weight (g/mol)	Num. H-Bond Donors	Num. H-Bond Acceptors	Num. Rotatable Bonds	TPSA	Consensus Log Po/w	GI Absorption	BBB Permeant	Solubility
64778	415.53	3	2	13	70.23 Å^2^	4.12	High	Yes	Poorly soluble
43932	486.15	2	6	8	98.56 Å^2^	3.25	High	No	Poorly soluble
41093	476.46	2	8	5	121.25 Å^2^	2.54	High	No	Moderately soluble
27363	406.41	4	5	6	121.30 Å^2^	1.57	High	No	Moderately soluble
42711	367.20	3	6	7	118.01 Å^2^	1.21	High	No	Moderately soluble

**Table 4:** ADME prediction results of 17 lead compounds obtained with small molecule 42711 after fragment substitution.

Molecule	Molecular Weight (g/mol)	Num. H-Bond Donors	Num. H-Bond Acceptors	Num. Rotatable Bonds	TPSA	Consensus Log Po/w
42711_10	400.23	2	8	7	128.25 Å^2^	1.33
42711_11	385.19	2	7	7	102.47 Å^2^	2.39
42711_13	369.19	4	7	7	128.07 Å^2^	0.64
42711_15	368.19	3	7	7	130.90 Å^2^	0.88
42711_21	370.18	3	8	7	130.10 Å^2^	0.38
42711_22	370.18	3	8	7	130.10 Å^2^	0.24
42711_24	385.26	3	6	7	127.63 Å^2^	1.87
42711_25	381.20	3	8	7	125.17 Å^2^	0.92
42711_33	387.16	2	9	7	128.25 Å^2^	1.49
42711_35	368.19	2	7	7	120.04 Å^2^	0.76
42711_36	368.21	4	4	7	101.59 Å^2^	0.94
42711_37	386.20	4	7	7	115.18 Å^2^	1.47
42711_38	368.18	2	7	7	115.36 Å^2^	1.39
42711_39	367.20	3	6	7	118.01 Å^2^	1.32
42711_40	368.21	4	6	7	115.18 Å^2^	1.00
42711_41	367.22	4	5	7	102.29 Å^2^	1.61
42711_42	367.19	2	6	7	102.47 Å^2^	1.83

**Table 5:** Interaction plots of positive compounds, compound 42711 and its fragment substitution products with target protein docking.

Molecule	2D Interaction Diagram	3D Interaction Diagram
Erastin	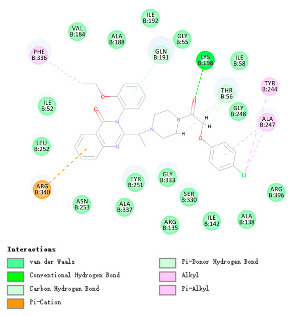	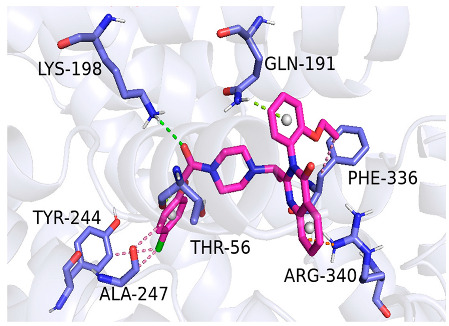
42711	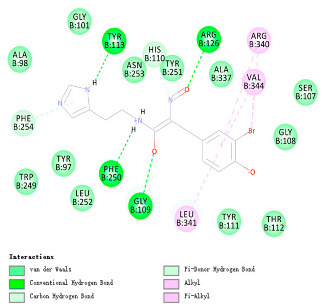	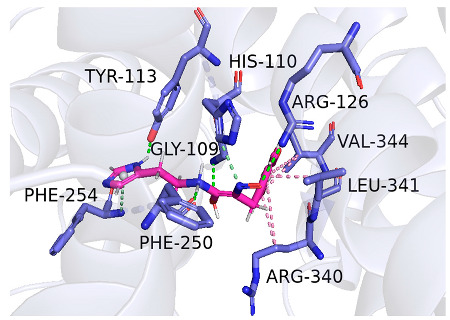
42711_11	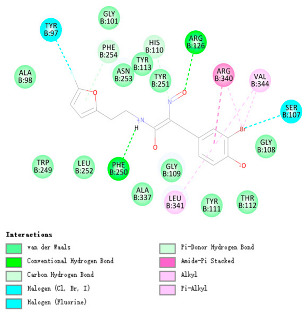	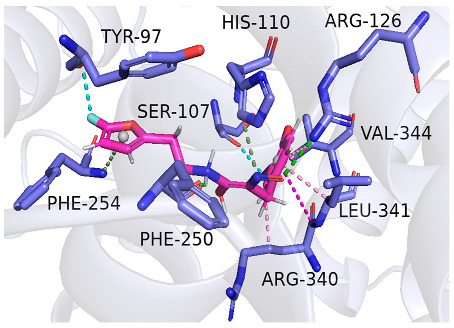
42711_38	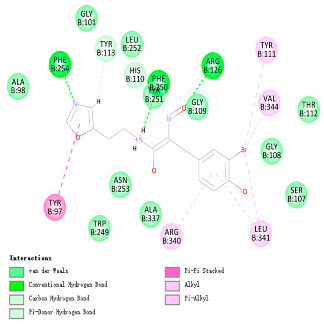	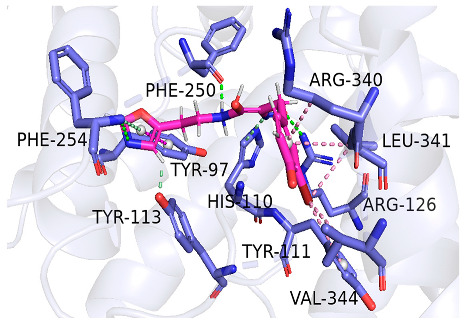

## Data Availability

The data used to support the findings of this study are included within the article.

## Supplementary Materials

The following supporting information can be downloaded at https://www.mdpi.com/article/10.3390/md22080375/s1: Table S1. Molecular descriptors computed for 2D-QSAR model; Table S2. Compound 421711_11 for 391 tumors and 47 normal human cell lines; Table S3. Cytotoxicity of compound 42711_11 against the NCI 60 tumor cell line panel; Table S4. Compound 42711_38 for 391 tumors and 47 normal human cell lines; Table S5. Cytotoxicity of compound 42711_38 against the NCI 60 tumor cell line panel; Table S6: The 31 inhibitors targeting SLC7A11 with IC50 values and their structures.
