# Supplementary material for: Identification of Marine-Derived SLC7A11 Inhibitors: Molecular Docking, Structure-Based Virtual Screening, Cytotoxicity Prediction, and Molecular Dynamics Simulation
Source: Mar Drugs. 2024 Aug 20;22(8):375. doi: 10.3390/md22080375 (PMC11355350; doi:10.3390/md22080375)
Supplement: Supplementary file 1 [file marinedrugs-22-00375-s001.zip › marinedrugs-3137069-supplementary.pdf]

Table S1. Molecular descriptors computed for 2D-QSAR model

**Calculated Properties**
ALogP,Molecular_Weight,Num_H_Donors,Num_H_Acceptors,Num_RotatableBonds,Num_Rings,Num_AromaticRings,Molecular_FractionalPolarSurfaceArea,ECFC_6, ECFP_6,EPFC_6

Table S2. Compound 42711 _ 11 for 391 tumors and 47 normal human cell lines

Pa	Pi	Cell-line	Description	Tissue/Organ	Type	IAP
0.942	0	NCI-H929	Plasma cell myeloma	Bone Marrow	Myeloma	0.972
0.323	0.269	A2780cisR	Cisplatin-resistant ovarian carcinoma	Ovarium	Carcinoma	0.838
0.309	0.017	HUVEC	Umbilical vein endothelial cell	Endothelium	Normal	0.897
0.167	0.02	WI-38	Embryonic lung fibroblast	Lung	Normal	0.943
0.095	0.063	HaCaT	Keratinocyte	Skin	Normal	0.967
0.064	0.041	UACC-903	Malignant melanoma	Skin	Melanoma	0.954
0.039	0.007	CCRF-CEM/ VCR-1000	T-cell leukaemia	Blood	Leukemia	0.989

Table S3. Cytotoxicity of compound 42711_11 against the NCI 60 tumor cell line panel

threshold	Pa	Pi	DTP NCI60 cell-line	Tissue of origin	Sex	Epithelial	Histology	Ploidy	p53	IAP
**10nm**	0.66	0.029	LE:HL-60(TB)	Leukemia	F	no	Pro myelocytic leukemia	2n+/-, Near-diploid 46+/- (35-57)	MT	0.819
	0.638	0.015	ME:SK-MEL-2	Melanoma	M	no	Malignant melanotic melanoma	4n-, Hypotetraploid (81-91)	WT	0.882
	0.628	0.028	LE:RPMI-8226	Leukemia	M	no	Myeloma	3n-, Hypotriploid (58-68)	WT	0.846
**100nm**	0.66	0.029	LE:HL-60(TB)	Leukemia	F	no	Pro myelocytic leukemia	2n+/-, Near-diploid 46+/- (35-57)	MT	0.819
	0.638	0.015	ME:SK-MEL-2	Melanoma	M	no	Malignant melanotic melanoma	4n-, Hypotetraploid (81-91)	WT	0.882
	0.628	0.028	LE:RPMI-8226	Leukemia	M	no	Myeloma	3n-, Hypotriploid (58-68)	WT	0.846

Table S4. Compound 42711_38 for 391 tumors and 47 normal human cell lines

Pa	Pi	Cell-line	Description	Tissue/Organ	Type	IAP
0.423	0.029	CCRF-CEM	Childhood T acute lymphoblastic leukemia	Blood	Leukemia	0.913
0.343	0.146	NCI-H358	Bronchioalveolar Carcinoma	Lung; Bronchiole	Carcinoma	0.805
0.224	0.152	NCI-H295R	Adrenal cortex carcinoma	Adrenal cortex	Carcinoma	0.856
0.214	0.071	HUVEC	Umbilical vein endothelial cell	Endothelium	Normal	0.897
0.205	0.149	CAPAN-1	Pancreas Adenocarcinoma	Pancreas	Adenocarcinoma	0.889
0.171	0.089	HT-1080	Fibrosarcoma	Soft tissue	Sarcoma	0.918
0.153	0.044	SJSA-1	Osteosarcoma	Bone	Sarcoma	0.94
0.136	0.037	WI-38	Embryonic lung fibroblast	Lung	Normal	0.943
0.131	0.116	A-431	Epidermoid carcinoma	Skin	Carcinoma	0.937
0.128	0.031	HaCaT	Keratinocyte	Skin	Normal	0.967
0.036	0.009	CCRF-CEM/VCR-1000	T-cell leukaemia	Blood	Leukemia	0.989

Table S5. Cytotoxicity of compound 42711_38 against the NCI 60 tumor cell line panel

threshold	Pa	Pi	DTP NCI60 cell-line	Tissue of origin	Sex	Epithelial	Histology	Ploidy	p53	IAP
**10nm**	0.788	0.013	LE:SR	Leukemia	M	no	Lymphoma	2n+/-, Near-diploid 46+/- (35-57)	NA	0.828
	0.317	0.104	LC:NCI-H460	Non-Small Cell Lung	M	yes	Large Cell Carcinoma-ud	2n+/-, Near-diploid 46+/- (35-57)	WT	0.874
**100nm**	0.788	0.013	LE:SR	Leukemia	M	no	Lymphoma	2n+/-, Near-diploid 46+/- (35-57)	NA	0.828
	0.317	0.104	LC:NCI-H460	Non-Small Cell Lung	M	yes	Large Cell Carcinoma-ud	2n+/-, Near-diploid 46+/- (35-57)	WT	0.874

Table S6. The 31 inhibitors targeting SLC7A11 with IC50 values and their structures

Compound	Structure	PIC50(nmol)
**CHEMBLE421**	O=C(O)c1cc(/N=N/c2ccc(S(=O)(=O)Nc3ccccn3)cc2)ccc1O	4.47712
**CHEMBLE700**	Nc1ccc(S(=O)(=O)Nc2ccccn2)cc1	6
**CHEMBLE28885**	N[C@@H](COS(=O)(=O)O)C(=O)O	4.41497
**CHEMBLE94990**	N[C@H](C(=O)O)c1ccc(C(=O)O)cc1	3.69897
**CHEMBLE230951**	N[C@@H](CCS(=O)(=O)O)C(=O)O	4.54407
**CHEMBLE401989**	CCOc1ccccc1-n1c(C(C)N2CCN(C(=O)COc3ccc(Cl)cc3)CC2)nc2ccccc2c1=O	2.30103
**CHEMBLE590556**	C/C(=N\Nc1ccc([N+](=O)[O-])cc1[N+](=O)[O-])c1c(C(=O)O)noc1C	5.4624
**CHEMBLE601837**	C/C(=N\Nc1ccc([N+](=O)[O-])cc1)c1c(C(=O)O)noc1C	5.1038
**CHEMBLE604574**	C/C(=N\Nc1cc(C(F)(F)F)cc(C(F)(F)F)c1)c1c(C(=O)O)noc1C	5.11059
**CHEMBLE606915**	C/C(=N\Nc1ccc([N+](=O)[O-])cc1[N+](=O)[O-])c1c(C(=O)O)noc1CCc1ccc2ccccc2c1	5.35218
**CHEMBLE1614646**	Cc1cccnc1NS(=O)(=O)c1ccc(C#Cc2ccc(O)c(C(=O)O)c2)cc1	4.8451
**CHEMBLE1834953**	COC(=O)c1cc(C#Cc2ccc(S(=O)(=O)Nc3ccccn3)cc2)ccc1O	6
**CHEMBLE1834954**	O=C(O)c1cc(C#Cc2ccc(S(=O)(=O)Nc3ccccn3)cc2)ccc1O	4.47712
**CHEMBLE1834955**	COC(=O)c1cc(/C=C/c2ccc(S(=O)(=O)Nc3ccccn3)cc2)ccc1O	5.95424
**CHEMBLE1834956**	O=C(O)c1cc(/C=C/c2ccc(S(=O)(=O)Nc3ccccn3)cc2)ccc1O	4.47712
**CHEMBLE1834957**	COC(=O)c1cc(CCc2ccc(S(=O)(=O)Nc3ccccn3)cc2)ccc1O	6
**CHEMBLE1834958**	O=C(O)c1cc(CCc2ccc(S(=O)(=O)Nc3ccccn3)cc2)ccc1O	5.8451
**CHEMBLE1834959**	O=C(O)c1cccc(C#Cc2ccc(S(=O)(=O)Nc3ccccn3)cc2)c1	4.87506
**CHEMBLE1834960**	O=C(O)c1cccc(CCc2ccc(S(=O)(=O)Nc3ccccn3)cc2)c1	5.96379
**CHEMBLE1834961**	O=C(O)c1cc(/N=N/c2ccccc2)ccc1O	5.30103

**CHEMBLE1834962**	O=C(O)c1cc(C#Cc2ccccc2)ccc1O	5.17609
**CHEMBLE1834963**	O=C(O)c1cccc(C#Cc2ccccc2)c1	5.43136
**CHEMBLE3629574**	CC(C)Oc1ccc(CN2CCNCC2)cc1-n1c(CN2CCN(C(=O)COc3ccc(Cl)cc3)CC2)nc2ccccc2c1=O	2.90309
**CHEMBLE3629575**	CC(C)Oc1ccc(C=O)cc1-n1c(CN2CCN(C(=O)COc3ccc(Cl)cc3)CC2)nc2ccccc2c1=O	1.77815
**CHEMBLE3629576**	CC(=O)c1ccc(OC(C)C)c(-n2c(CN3CCN(C(=O)COc4ccc(Cl)cc4)CC3)nc3ccccc3c2=O)c1	1.47712
**CHEMBLE3629577**	CC(C)Oc1ccc(C(=O)CF)cc1-n1c(CN2CCN(C(=O)COc3ccc(Cl)cc3)CC2)nc2ccccc2c1=O	1.60206
**CHEMBLE3629578**	CC(C)Oc1ccc(C(=O)C(F)(F)F)cc1-n1c(CN2CCN(C(=O)COc3ccc(Cl)cc3)CC2)nc2ccccc2c1=O	1.30103
**CHEMBLE3629579**	CC(C)Oc1ccc(C(=O)CN2CCOCC2)cc1-n1c(CN2CCN(C(=O)COc3ccc(Cl)cc3)CC2)nc2ccccc2c1=O	1
**CHEMBLE3629580**	CC(C)Oc1ccc(C(=O)CN2CCN(C)CC2)cc1-n1c(CN2CCN(C(=O)COc3ccc(Cl)cc3)CC2)nc2ccccc2c1=O	2.47712
**CHEMBLE3629669**	COc1ccc(CN2CCN(CC(=O)c3ccc(OC(C)C)c(-n4c(CN5CCN(C(=O)COc6ccc(Cl)cc6)CC5)nc5ccccc5c4=O)c3)CC2)cc1	0.60206
**CHEMBLE3629671**	CC(C)Oc1ccc(C(=O)Cn2ccnc2)cc1-n1c(CN2CCN(C(=O)COc3ccc(Cl)cc3)CC2)nc2ccccc2c1=O	1.47712

.
